# Generation of Gellan Gum-Based Adipose-Like Microtissues

**DOI:** 10.3390/bioengineering5030052

**Published:** 2018-06-27

**Authors:** Manuela E. L. Lago, Lucília P. da Silva, Catarina Henriques, Andreia F. Carvalho, Rui L. Reis, Alexandra P. Marques

**Affiliations:** 1 3B’s Research Group—Biomaterials, Biodegradables and Biomimetics, Headquarters of the European Institute of Excellence on Tissue Engineering and Regenerative Medicine, University of Minho, Avepark, Barco, 4805-017 Guimarães, Portugal; 2 ICVS/3B’s—PT Government Associate Laboratory, 4806-909 Braga/Guimarães, Portugal; 3 The Discoveries Centre for Regenerative and Precision Medicine, Headquarters at University of Minho, 4704-553 Guimarães, Portugal

**Keywords:** adipose tissue engineering, adipogenic differentiation, adipose-like microtissues, gellan gum, hydrogels

## Abstract

Adipose tissue is involved in many physiological processes. Therefore, the need for adipose tissue-like analogues either for soft tissue reconstruction or as in vitro testing platforms is undeniable. In this work, we explored the natural features of gellan gum (GG) to recreate injectable stable adipose-like microtissues. GG hydrogel particles with different percentages of polymer (0.5%, 0.75%, 1.25%) were developed and the effect of obtained mechanical properties over the ability of hASCs to differentiate towards the adipogenic lineage was evaluated based on the expression of the early (PPARγ) and late (FABP4) adipogenic markers, and on lipids formation and accumulation. Constructs were cultured in adipogenic induction medium up to 21 days or for six days in induction plus nine days in maintenance media. Overall, no significant differences were observed in terms of hASCs adipogenic differentiation within the range of Young’s moduli between 2.7 and 12.9 kPa. The long-term (up to six weeks) stability of the developed constructs supported its application in soft tissue reconstruction. Moreover, their ability to function as adipose-like microtissue models for drug screening was demonstrated by confirming its sensitivity to TNFα and ROCK inhibitor, respectively involved in the repression and induction of the adipogenic differentiation.

## 1. Introduction

Adipose tissue is no longer considered solely an energy storage tissue. It is a complex tissue involved in several biological processes including the endocrine/paracrine regulation of energy metabolism and thermoregulation, that also provides key structural protection and support to major organs [1,2]. Increasing adipose tissue loss associated with various pathological conditions, namely oncologic resection, trauma, and congenital abnormalities, has been supporting the need for improved strategies for soft tissue reconstruction, currently limited due to high resorption rates [3]. Moreover, endocrine and metabolic diseases such as diabetes and obesity, hallmarks of developed societies, require additional knowledge to be delivered for further improving the targeted therapeutics and enhanced success rates. Considering this, new strategies to generate adipose-like tissues that can be either used in soft tissue reconstruction or as reliable in vitro models are in an undeniable demand. 

The combination of adult stem cells from various sources within 3D polymeric structures has been one of the most approached to generate 3D adipose-like tissues. The majority of these works have been using 3T3-L1 [4,5,6,7], a murine pre-adipocyte cell line that has limited representation of human tissue physiology. In alternative, adult stem cells have been differentiated into the adipogenic lineage in a range of natural/synthetic materials [8,9,10,11]. Considerations, such as the mimicking of the dimensions of the fat lobules at the microscale [8] and of adipose tissue mechanical properties, have been addressed from the material’s perspective at the initial stage of development. While these have been shown to correlate with the achieved degree of differentiation, it is also known that to be reliable and functional, the generated constructs have to possess appropriate stability. Factors such as the type of material [10,12,13] and respective degradation rate, in vitro culture conditions [11,14] and (pre)-vascularization [8,11] are among those known to influence it however, little is known about the requirements to achieve ideal stability.

Gellan gum (GG) is a natural polymer that has been proposed for various tissue engineering and regenerative medicine applications [15,16,17,18]. Because GG is thermosensitive and reacts with monovalent or divalent cations, it forms hydrogels by temperature change and by ionic crosslinking. Thus, the properties of GG hydrogels are highly tunable by changing the polymer and/or crosslinking concentration, or even crosslinking type and conditions. Moreover, GG is not susceptive to enzymatic action which makes GG hydrogels degradation dependent on hydrolytic reactions. Cell adhesiveness, except when combined with peptide sequences [19,20] or processed in particular modes [21,22], is not a feature naturally depicted by GG hydrogels.

**Scheme 1:**
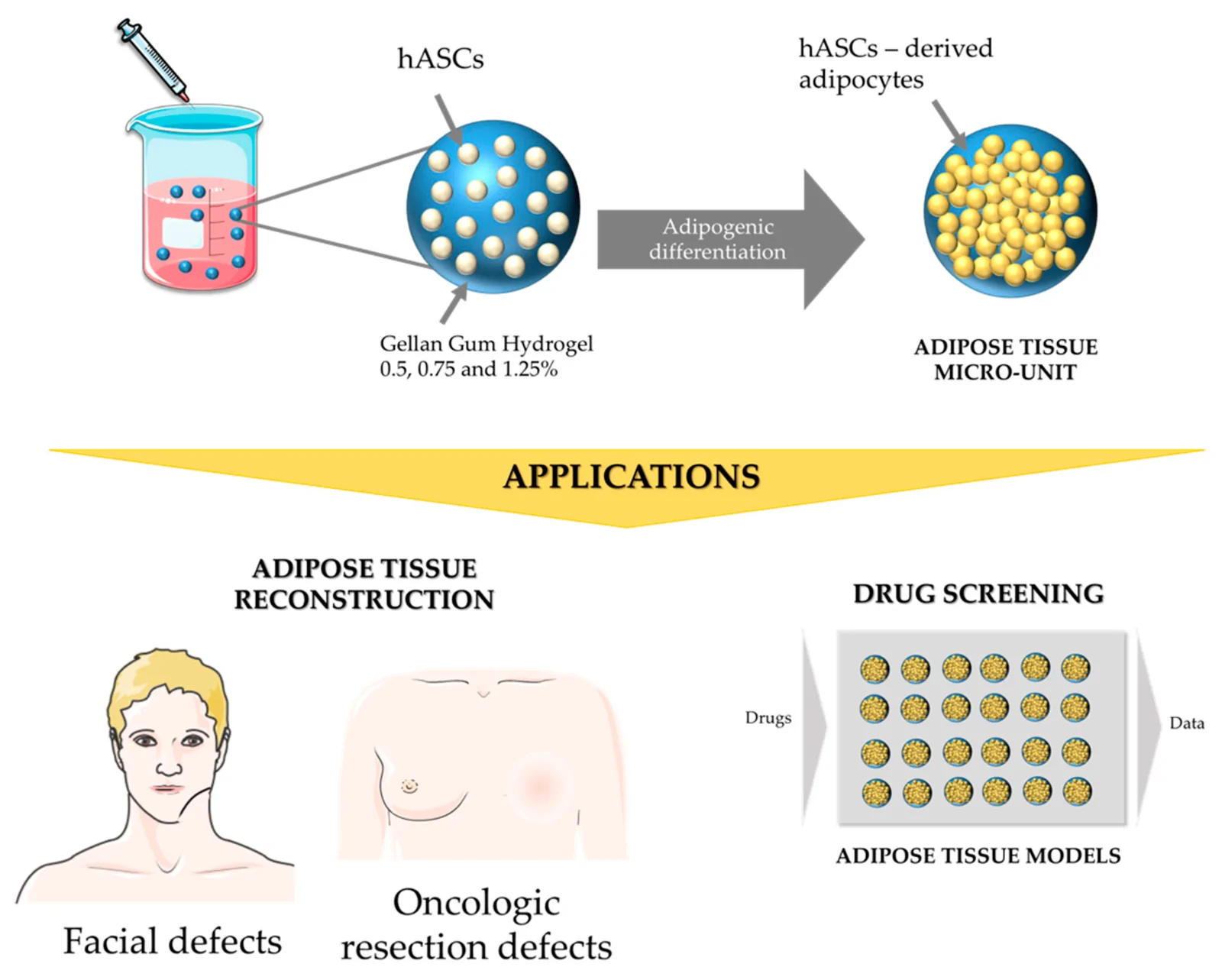
Schematic representation of the development of adipose-like microtissues and potential applications. Human adipose derived stem cells (hASCs) are encapsulated in gellan gum-based hydrogel particles with different percentages of polymer, and differentiated into the adipogenic lineage. The obtained adipose-like microtissues can be used for adipose tissue reconstruction of defects from oncologic resection, trauma and congenital abnormalities, or as 3D in vitro tissue analogues for drug screening.

Considering that along adipogenic differentiation, cells suffer continuous alterations in integrin expression and that disruption of the extracellular matrix (ECM)-cell contact is fulcra [1], we hypothesized that natural GG hydrogels features, like reduced cell-ECM interactions mimicking and degradation rate, would potentiate adipogenic differentiation of human adipose derived stem cells (hASCs). Moreover, by tuning the physic-chemical and mechanical properties of GG hydrogels we could add to the generation of stable GG-based adipose-like microtissues. Hence, GG hydrogel particles with different percentages of polymer were developed and the effect of obtained mechanical properties over the ability of hASCs to differentiate towards the adipogenic lineage was evaluated. Degradability was also assessed to assure the stability of the developed constructs, critical for its application in soft tissue reconstruction. Moreover, their ability to function as adipose-like microtissue models for drug screening was analysed by testing its sensitivity to molecules involved in the induction/repression of the adipogenic differentiation (Scheme 1).

## 2. Materials and Methods

### 2.1. Gellan Gum Hydrogel Particles Preparation

Gellan gum (GG) hydrogels were prepared as previously described [21] with some modifications. Gelzan (Sigma-Aldrich, Saint Louis, MI, USA) was filtered through a sterile filtration system (Techno Plastic Products AG, Trasadingen, Switzerland), frozen in liquid nitrogen and lyophilized (LyoAlfa 10/15, Telstar, Terrassa, Spain) for three days to obtain sterile, dried and purified material. This was dissolved in deionized (DI) H_2_O to attain a final concentration of 0.5%, 0.75%, and 1.25% (*w*/*v*). After complete dissolution of the polymer at 90 °C under stirring, the temperature was lowered to 40 °C and Alpha Minimum Essential Medium (α-MEM) (Life Technologies, Paisley, UK) was added and then dispensed into a hydrophobic surface using a syringe/pipette for viscous solutions to produce spherical hydrogel particles. The size of the particles was given by the dispensed amount (1 to 50 µL). The injectability was assessed by coupling a 27 G needle in a syringe (1 mL) by loading the particles in a GG vehicle solution. Images of the particles were acquires using a Stereo Microscope + Lamp (Schott KL 200, Stemi 1000, Carl Zeiss, Oberkochen, Germany) combined with AxioVision software (version 4.8.2.0, Carl Zeiss, Oberkochen, Germany) which allows to measure hydrogel particles diameter.

### 2.2. Oscillatory Rheology

The viscoelastic properties were determined using an oscillatory rheometer (MAL1097376, Kinexus Prot, Malvern, UK). Hydrogels discs (5 mm Ø, 4 mm height) were prepared as previously described for the particles but using a punctured mould and maintained in complete α-MEM medium for two days to reach the swelling equilibrium. The discs were loaded in the rheometer between parallel plates (25 mm in diameter) adjusting the gap to reach a normal force close to 0 N (normal force maximum is 0.5 N). The storage (G′) and loss (G′′) moduli were measured by performing a dynamic time sweep at a frequency of 10 rad/s, 0.5% strain and at 25 °C along time until a plateau was reached. 

### 2.3. Degradation

The degradation of GG hydrogel particles was analysed by determining the mass loss after incubation with a NaCl (0.154 M) solution at 37 °C and stirring (180 rpm) for 42 days. Mass loss was determined by measuring the weight of GG hydrogels particles before (Wi) and after (Wf), immersion into the NaCl solution. Maximum mass loss at the end of the incubation time (six weeks) was also determined by measuring the weight of the dried GG hydrogels particles after freeze-drying at the final time-point.

Mass loss was calculated according to the following equation:

$$\mathrm{mass}\left(\%\right)=\frac{\mathrm{Wf}-\mathrm{Wi}}{\mathrm{Wi}}\times 100$$

where Wi represents the initial weight of the hydrogel particles and Wf represents the weight of the hydrogel particles at each time-point or the dried hydrogel particles weight at the last time-point after freeze-drying.

### 2.4. Isolation and Characterization of Human Adipose Stem Cells

Human adipose stem cells (hASCs) were isolated as previously described [23] from the subcutaneous adipose tissue of three donors that underwent liposuction procedures at Hospital da Prelada (Porto). Samples were collected with the informed consent of the patients and under a collaboration protocol with 3B’s Research Group, approved by the ethical committees of both institutions. hASCs were expanded in Alpha Minimum Essential Medium (α-MEM) supplemented with 10% Foetal Bovine Serum (FBS) (Life Technologies, Bleiswijk, The Netherlands) and 1% Antibiotic/Antimycotic (ATB) (Life Technologies, Paisley, UK) in a humidified atmosphere with 5% CO_2_ at 37 °C. The mesenchymal stem cell phenotype of hASCs was confirmed by the expression of the principal mesenchymal markers (CD90, CD105 and CD73) analysed by flow cytometry and after differentiation into the adipogenic and osteogenic lineages (Supplemental Material). 

### 2.5. Cell Encapsulation within Gellan Gum Hydrogels Particles

Human adipose stem cells were resuspended in GG solution at concentrations of 3 × 10^3^ cells/µL. After proper dispersion of the cells within the polymeric solution, hydrogel particles were prepared as described above. Cell-laden hydrogel particles were incubated in supplemented α-MEM medium in a humidified atmosphere with 5% CO_2_ at 37 °C. 

### 2.6. Adipogenic Differentiation within Gellan Gum Hydrogels Particles

Adipogenic differentiation of hASCs within gellan gum hydrogel particles was induced as previously described [24] with some modifications. After culturing cell-laden hydrogel particles in α-MEM medium for three days, medium was changed to an adipogenic induction medium (IM) consisting of α-MEM supplemented with 10% FBS, 1% ATB, 34 µM of D-pantothenate (Sigma-Aldrich, Sintra, Portugal) and 66 µM of biotin (Sigma-Aldrich, Sintra, Portugal), 200 nM of insulin (Sigma-Aldrich, Sintra, Portugal), 1 µM of dexamethasone (Sigma-Aldrich, Sintra, Portugal), 250 µM of 3-isobutyl-1-methylxanthine (IBMX) (Sigma-Aldrich, Sintra, Portugal), and 5 µM of troglitazone (Sigma-Aldrich, Sintra, Portugal). Cells were cultured for further 3, 6, 15 and 21 days. An additional condition was set after days six of induction, by changing the medium to maintenance medium (MM)—IM without IBMX and troglitazone—and culture the cell-laden hydrogel particles for a further nine days. 

### 2.7. Incubation with TNF Alpha and ROCK Inhibitor

To assess the influence TNF alpha (inhibitor) and Rho kinases (ROCK) inhibitor (promoter) on the adipogenic differentiation, 0.5% GG hydrogel particles with hASCs were cultured for 15 days in induction medium supplemented with 0.5 µg/mL of TNFα or 50 µM of ROCK inhibitor. Control conditions in basal medium and in differentiation medium without the tested molecules were considered.

### 2.8. Quantitative Real Time Polymerase Chain Reaction (RT-PCR)

Cell-laden hydrogel particles were collected in 400 µL of Tri-reagent (Sigma-Aldrich, Sintra, Portugal) and preserved at −80 °C until extraction. For RNA extraction, samples were incubated for 5 min at RT, macerated with a tissue grinder (Nippon genetics, Duren, Germany) and subsequently centrifuged for 5 min at 6000 G. The pellet was discarded and 80 µL of chloroform (Sigma-Aldrich, Sintra, Portugal) was added to the recovered supernatants. Following an incubation period of 15 min at RT, samples were centrifuged at 4 °C for 20 min at 13,000 rpm. After centrifugation, the aqueous phase was collected and 200 µL of isopropanol (VWR, Carnaxide, Portugal) were added. Following a 10 min of incubation period at RT, samples were centrifuged at 4 °C for 10 min at 13,000 rpm. The supernatants were discarded and pellets were washed once with 100% ethanol and twice with 70% ethanol, by centrifugation at 4 °C for 5 min at 13,000 rpm. Extracted RNA was kept in 10 µL of RNAse/DNAse free water (Lonza, Verviers, Belgium). 

RNA quantity and purity were assessed using a NanoDrop N-1000 Spectrophotometer (Thermo Fischer Scientific, Wilmington, DE, USA). Samples with a 260/280 nm ratio between 1.6 and 2.2 were used for cDNA synthesis. Synthesis was performed using a QSCript cDNA SuperMix (Quanta Biosciences, Gaithersburg, MD, USA) and a Reverse Transcription Polymerase Chain Reaction (RT-PCR) Mastercycler (Eppendorf, Hamburg, Germany). An initial amount of 200 ng of RNA in RNAse/DNAse free water was used for a total volume of 20 µL.

FABP4 and PPARγ transcripts were quantified in the cDNA samples using a Quantitative Real Time Polymerase Chain Reaction (qRT-PCR). The primers were designed using the Primer-BLAST tool (NCBI, Bethesda, MD, USA) and synthesized by Eurofins genomics (Ebersberg, Germany) as listed in Table 1. The Real-Time PCR reaction was done using PerfeCta SYBR Green FastMix (Quanta Biosciences, Gaithersburg, MD, USA), following manufacturer’s instructions in a Reverse Transcription Polymerase Chain Reaction Mastercycler (Eppendorf, Hamburg, Germany). FABP4 and PPARγ were amplified for 45 cycles in a total volume of 20 µL. Each cycle comprised a denaturation step at 95 °C for 10 s, followed by an annealing step at specific temperatures (see Table 1) for 30 s and an extension step at 72 °C for 30 s. Relative gene expression was analysed against GAPDH housekeeping gene.

### 2.9. Western Blot

Cell-laden hydrogels were collected in radio-immunoprecipitation assay (RIPA) buffer containing protease inhibitor (1:100) and Dithiothreitol (DTT) (1:1000), all from Sigma-Aldrich (Sintra, Portugal), to reduce disulphide bonds and protect proteins from denaturation. Samples were macerated with a tissue grinder (Nippon genetics, Duren, Germany), left for 30 min in lysis buffer solution and centrifuged for 20 min at 4 °C and 12,000× *g*. Protein quantification was performed using Bradford assay kit (Thermo Fisher Scientific, Rockford, IL, USA), according to manufacturer’s instructions. For Western Blot, 10 µg of each samples were loaded in a 19% SDS polyacrylamide gel (Sigma-Aldrich, Sintra, Portugal) for electrophoresis and subsequently transferred onto a nitrocellulose membrane (GE Healthcare, Buckinghamshire, UK). The blot was then incubated in Ponceau solution, and posteriorly blocked with a 5% milk powder solution for 1 h. The blot was incubated at 4 °C overnight with a rabbit polyclonal FABP4 antibody (1:1000) and with a rabbit polyclonal beta-tubulin antibody (Abcam, Cambridge, UK) at a dilution of 1:500 (loading control). The bound antibody was detected with an anti-rabbit alkaline phosphatase secondary antibody (Sigma-Aldrich, Sintra, Portugal) diluted in a 5% milk powder solution at 1:5000. Bands were visualized using an AP Conjugate Substrate kit (Biorad, Lisbon, Portugal), followed by scanning with an EPSON Perfection V600 Scanner (EPSON, Nagano, Japan). Band intensities were quantified using ImageJ software (Version 1.52b, NIH, Baltimore, MD, USA).

### 2.10. Immunocytochemistry

Cell-laden hydrogel particles were fixed in 10% *v*/*v* of formalin (Bio-Optic, Milano, Italy) for 1 h, incubated with 1% Triton X-100 (Sigma-Aldrich, Sintra, Portugal) for 20 min at 4 °C, and with 2.5% *w*/*v* of horse serum (HS, Vector Laboratories, Burlingame, CA, USA) for 1 h, respectively for cell permeabilization and blocking of nonspecific antibody binding. Samples were then incubated with rabbit anti-human primary antibodies FABP4 (1:100), PPAR gamma (1:25) (Abcam, Cambridge, UK) diluted in 1% BSA, 0.2% Triton in PBS for 24 h. After washing with PBS, samples were incubated overnight at 4 °C with the secondary antibody Alexa Fluor 488 donkey anti-rabbit (Life Technologies, Carlsband, CA, USA) at a concentration of 1:500 in 1% HS in PBS. Nuclei were counter-stained with DAPI (0.02 mg/mL). Cell-laden hydrogel particles were observed using a Leica TCS SP8 confocal microscope (Leica, Mannheim, Germany) or an AxioImager Z1m fluorescence microscope (Zeiss, Gottingen, Germany).

### 2.11. Nile Red Staining and Quantification

Cell-laden hydrogel particles, previously fixed in 10% formalin, were placed in a diluted solution of Nile Red (0.05 µg/mL, 1:2000 dilution in PBS) and incubated for 20 min at 4 °C. Nuclei were counter-stained with DAPI (0.02 mg/mL). Lipids were observed using a Leica TCS SP8 confocal microscope (Leica, Mannheim, Germany) or an AxioImager Z1m fluorescence microscope (Zeiss, Gottingen, Germany). ImageJ software was used to count the number of Nile Red stained cells in relation to the total number of cells (DAPI) in 9 random images for each condition. 

### 2.12. Statistical Analysis

GraphPad Prism 7 software (La Jolla, CA, USA) was used to perform statistical analysis. The results were compared to a control condition corresponding to the samples before induction of the differentiation. Data was analysed using the Shapiro-Wilk normality test. Data following a normal distribution was analysed using a two-way ANOVA with Turkey post-test and a one-way ANOVA with Turkey post-test. Significance was set to * *p* < 0.05, *** *p* < 0.001; **** *p* < 0.0001). All quantitative data refer to *n* = 3 and are presented as mean ± standard deviation. 

## 3. Results

### 3.1. GG Hydrogel Particles Properties

**Figure 1:**
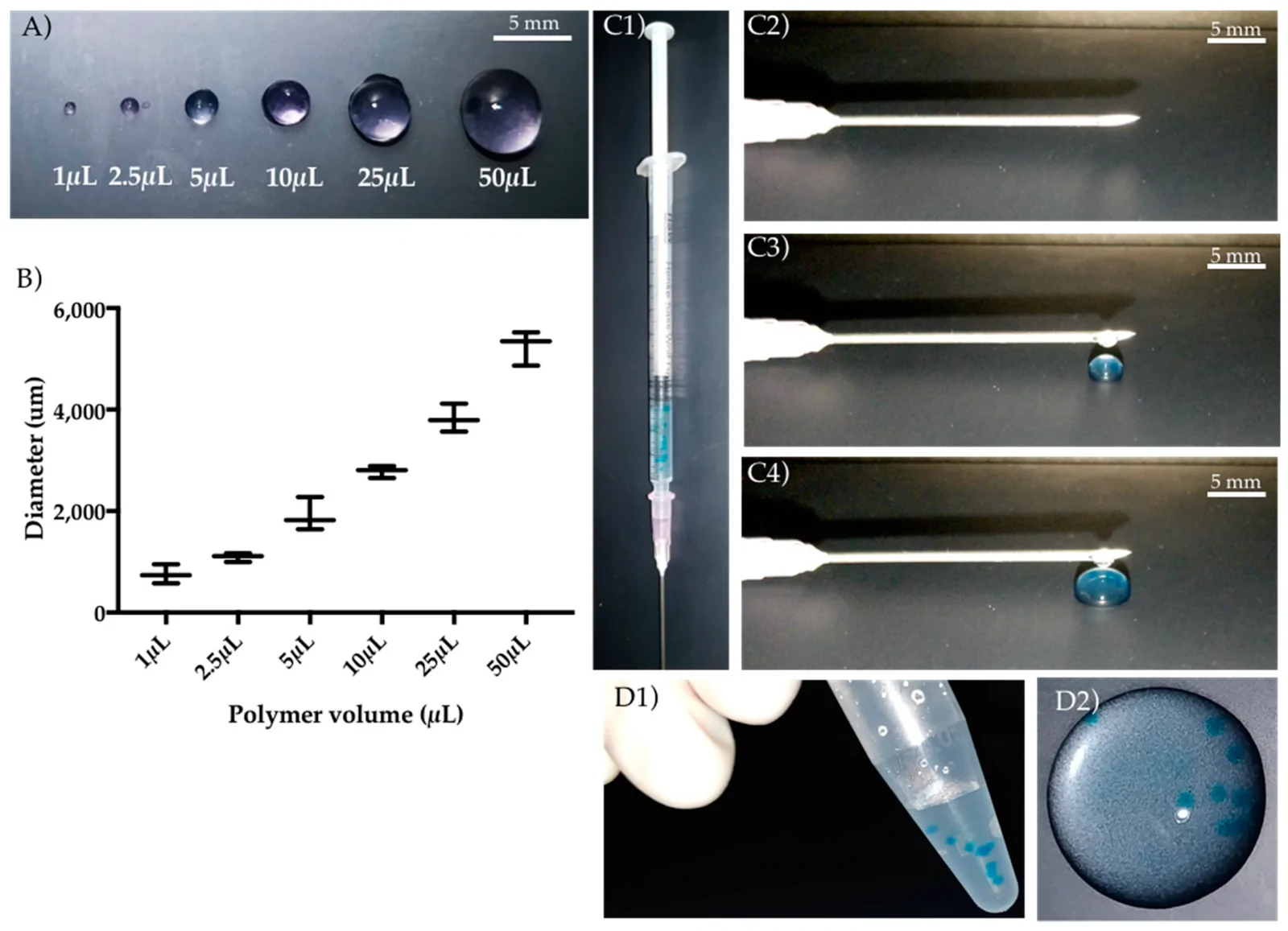
Production and injectability of gellan gum (GG) hydrogel particles. (**A**,**B**) Spherical particles with different sizes ranging from 700 to 5000 µm of diameter, approximately are obtained by dispensing different volumes of polymer solution (from 1 to 50 µL). (**C**) The obtained particles (previously stained with methylene blue) are loaded in a GG solution (**C1**) that acts as vehicle to confirm its injectability through a needle of 27G (**C2**–**C4**). (**D**) The integrity of the particles before (**D1**) is maintained after injection (**D2**).

Considering soft-tissue reconstruction and the possibility to inject the adipose tissue-like microtissues to fill in a defect, GG spherical hydrogel particles of different sizes were produced. The size of the particles, given by the dispensed amount (from 1 to 50 µL) (Figure 1A), ranged from 700 to 5000 µm in diameter, approximately (Figure 1B). To demonstrate that injection would not interfere with the integrity of the hydrogel particles, they were loaded in a GG vehicle solution and dispensed using a syringe (1 mL) coupled to a 27 G needle (Figure 1C). After injection the hydrogel particles kept the spherical shape and did not disintegrate (Figure 1C,D).

**Figure 2:**
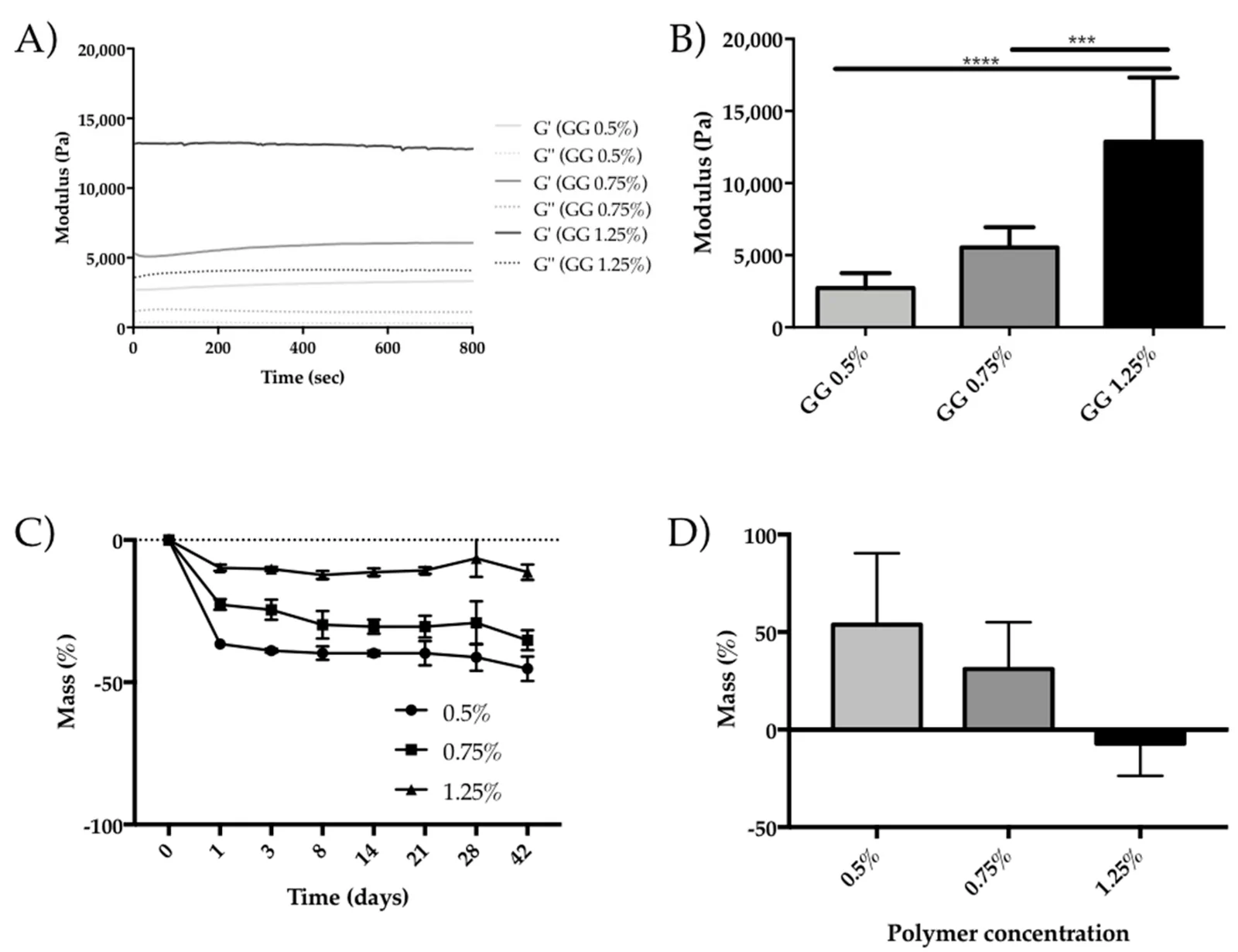
Physic-chemical and mechanical properties of gellan gum (GG) hydrogels. (**A**) Representative dynamic time sweep of storage (G′) and loss (G′′) moduli of GG hydrogels using strain of 0.5%, frequency of 10 rad/s and at 25 °C. (**B**) Plot of storage (G′) and loss (G′′) moduli of GG hydrogels obtained from the dynamic time sweep (*n* = 5). (**C**) Mass loss profile of GG hydrogel particles along incubation with a saline solution for 42 days (six weeks, *n* = 5). (**D**) Plot of maximum mass value after six weeks. *** *p* < 0.001 and **** *p* < 0.0001.

To determine if the amount of polymer affected the mechanical properties of the hydrogel particles, the storage (G′′) and loss (G′′) moduli were measured (Figure 2A). The storage modulus determined by the most linear curve in a dynamic time sweep (Figure 2B) was higher for hydrogels with 1.25% of polymer content, which represents an increase of 17% and 33% in comparison to hydrogels respectively with 0.75% (*p* < 0.05) and 0.5% (*p* < 0.05) of polymer content. 

The stability of the prepared GG hydrogel particles in saline solution was analysed based on the hydrogel particles mass loss up to 42 days (six weeks). After a fast mass loss within the first day, the profiles of the hydrogel particles kept relatively constant along the six weeks of incubation time (Figure 2C). When the dry weight of the particles was measures at the end time point, it was possible to see that the particles did not degrade and that the weight variations did not correspond to loss of polymeric content (Figure 2C,D). 

### 3.2. Assessment of hASCs Differentiation within GG Hydrogel Particles

To evaluate the influence of the physic-chemical and mechanical properties of the different formulations of GG hydrogel particles on the differentiation of hASCs, the expression of the early and late adipogenic markers, respectively PPARγ and FABP4, and of the lipid formation and accumulation were evaluated.

**Figure 3:**
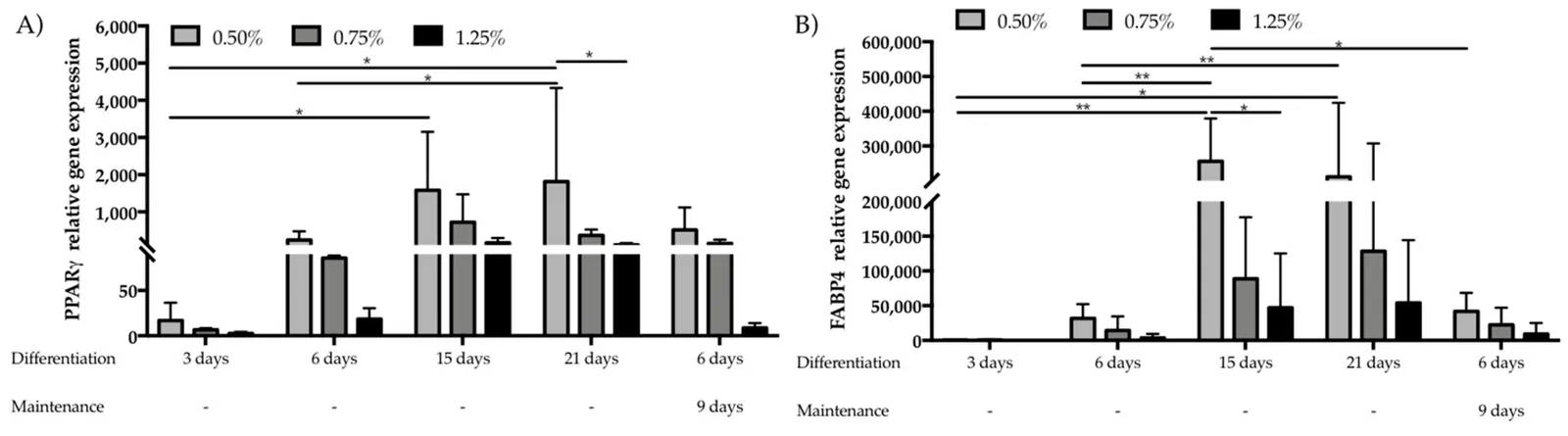
Relative expression of (**A**) PPARγ and (**B**) FABP4 genes in human adipose-derived stem cells within gellan gum hydrogel particles with different amounts of polymer (0.5%, 0.75% and 1.25%) cultured in adipogenic induction medium for 3, 6, 15 and 21 days and for six days plus maintenance medium for 9 days. * *p* < 0.05 and ** *p* < 0.01.

The results obtained from the qPCR analysis for PPARγ (Figure 3A) and FABP4 (Figure 3B) demonstrated that these genes started to be expressed after six days of culture in induction medium. The expression of PPARγ increased for higher times of culture in induction medium up to day 15. The switch to maintenance medium after six days of culture in induction one, did not affect the expression of PPARγ. In opposition, PPARγ expression within 0.5% GG hydrogel particles significantly increased (*p* < 0.05) along the time of culture in induction medium. This tendency seems to be also observed within 0.75% and 1.25% GG particles up to day 15, although without significant differences. The expression of FABP4 in the 0.5% GG hydrogel particles showed the same trend as PPARγ, significantly increasing (*p* < 0.05) along the time of culture in induction medium. Moreover, at day 15 the expression of PPARγ in the 0.5% GG hydrogel particles was significantly higher (*p* < 0.05) than in the 1.25% ones. Interestingly, independently of the concentration of the particles, the expression levels at day six in induction plus nine days in maintenance media were similar to those observed at day six only in induction medium, and significantly lower than those at day 15 within the 0.25% GG hydrogel particles. 

**Figure 4:**
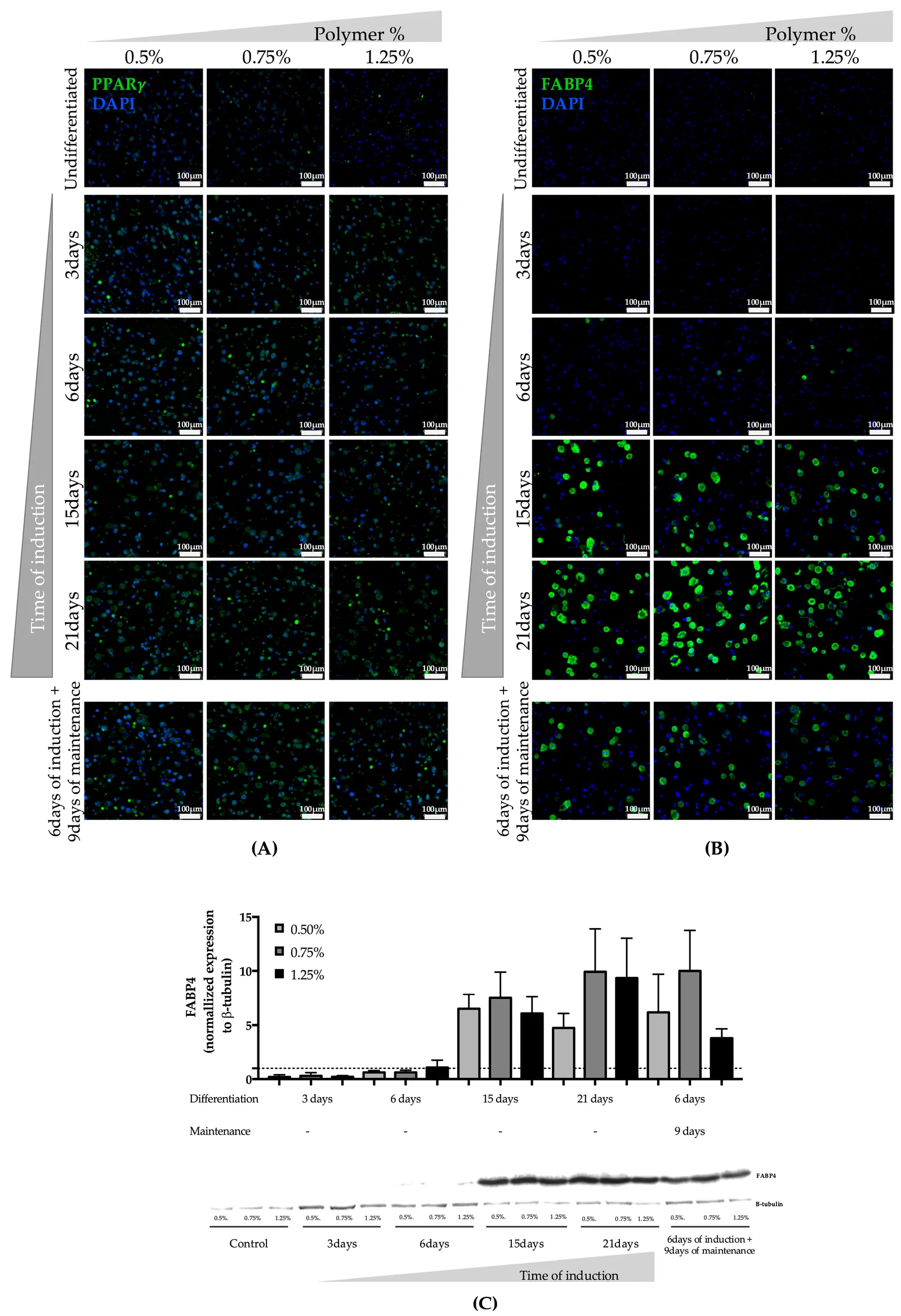
Adipogenic differentiation of human adipose-derived stem cells within gellan gum hydrogel particles with different amounts of polymer along the culture in adipogenic induction medium and after being cultured for six days in induction plus nine days in maintenance media. Representative confocal images of (**A**) PPARγ and (**B**) FABP4 expression (green). Cell nuclei were labelled with DAPI (blue). (**C**) Representative Western blot analysis of the expression of FABP4. Plotted data was normalized against beta-tubulin expression, which was used as a loading control. Scale bar: 100 µm.

To confirm the gene expression results and the hASCs capacity to differentiate towards adipogenic lineage within the GG hydrogel particles, the expression of PPARγ (Figure 4A) and FABP4 (Figure 4B) was evaluated at the protein level by immunocytochemistry. PPARγ expression started at day three and seemed to increase along the differentiation time (Figure 4A). The expression FABP4 was not detected until day 15 of culture in induction medium or after 6 days of culture in induction plus nine days in maintenance media. In comparison to day 15 of induction, it seems that a higher number of cells is expressing FABP4 at day 21 (Figure 4B). This trend was confirmed by Western blot (Figure 4C), although the semi-quantitative analysis did not reveal significant differences. 

**Figure 5:**
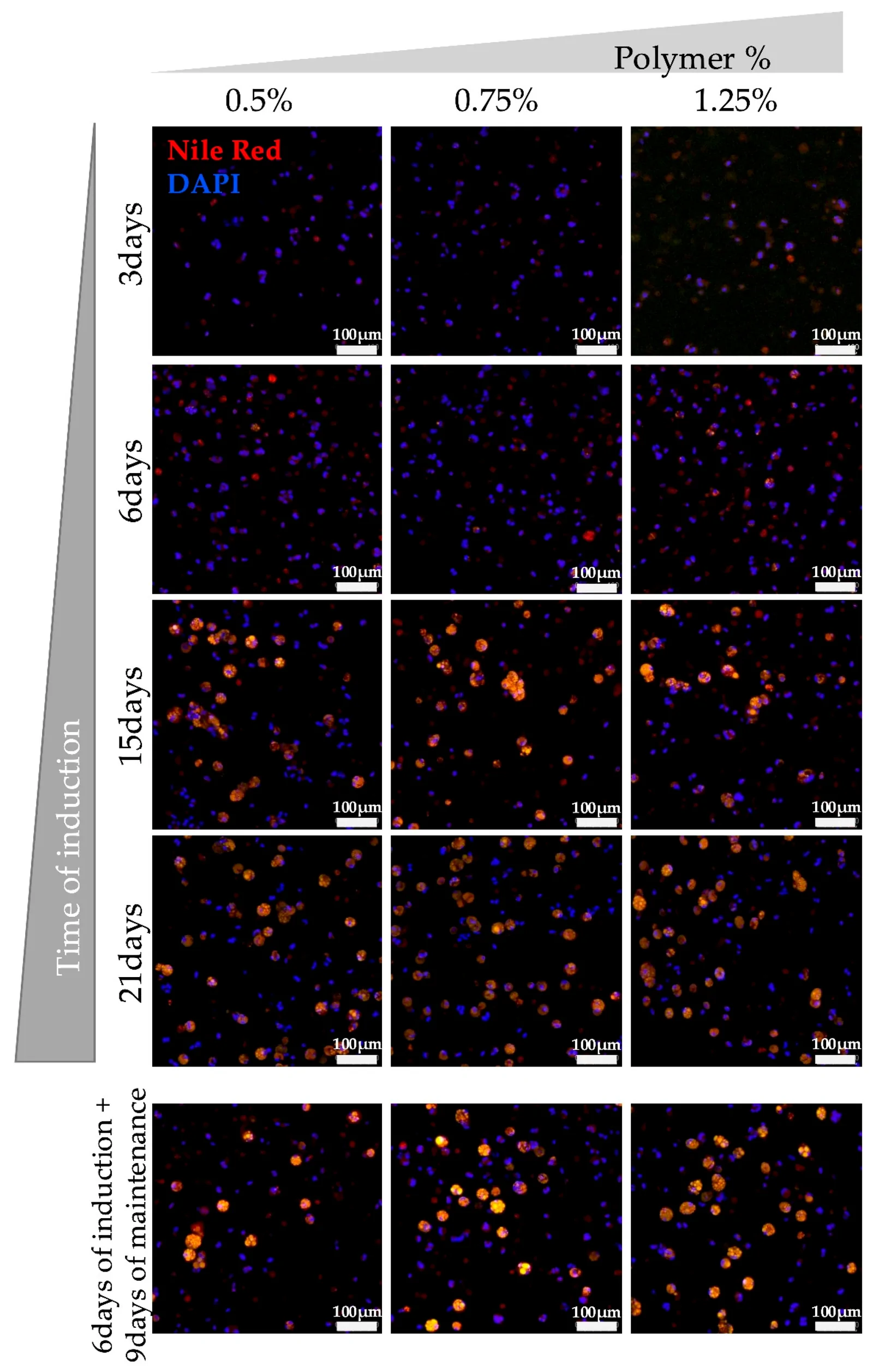
Neutral intracellular lipid accumulation in human adipose-derived stem cell-derived adipocytes within gellan gum hydrogel particles with different amounts of polymer along the culture in adipogenic induction medium and after being cultured for six days in induction plus nine days in maintenance media. Representative confocal images of cells stained with Nile red (yellow). Nuclei were labelled with DAPI (blue). Polar cell membrane lipids are stained in red. Scale bar: 100 µm.

To further confirm the adipogenic phenotype of the cells along the differentiation within the produced hydrogel particles, the accumulation of neutral intracellular lipids was analysed through Nile Red staining (Figure 5). These lipids started to be seen at day six of induction medium and accumulate at higher amounts at longer induction time-points. Moreover, when maintenance medium was added after the six days of culture in induction medium, the levels of neutral lipids seem to be similar to those observed after 15 days of culture in induction medium.

### 3.3. Assessment of TNF Alpha and ROCK Inhibitor Effect on Adipogenic Differentiation

**Figure 6:**
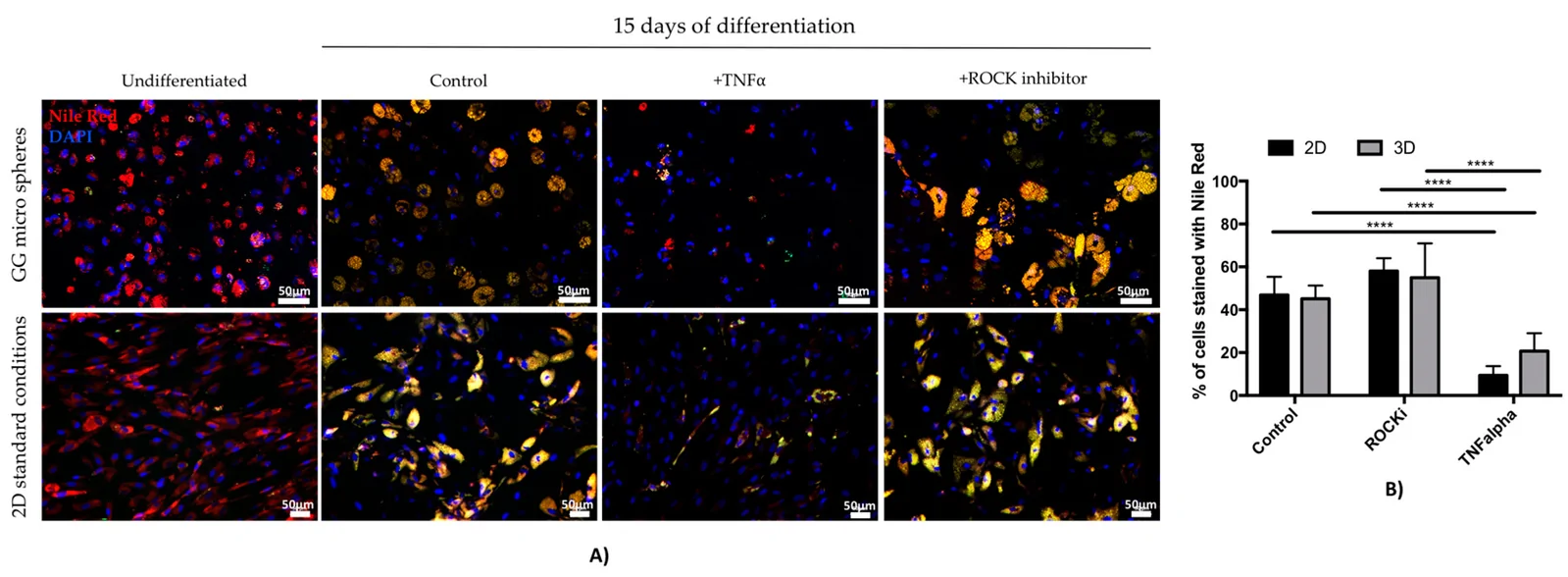
Assessment of the influence of TNFα (0.5 µg/mL) and ROCK inhibitor (50 µM) over the adipogenic differentiation of human adipose-derived stem cells within 0.5% gellan gum hydrogel particles after 15 days of culture in induction medium. (**A**) Representative images of nile red staining (polar cell membrane lipids are stained in red), and respective (**B**) quantification of the percentage of stained cells in relation to the total number of cells. Scale bar 50 µm. **** p < 0.0001.

In order to prove the responsive behaviour of the proposed systems, the induction medium was supplemented with TNFα or ROCK inhibitor during the adipogenic differentiation (Figure 6A). Nile Red staining confirmed that the differentiation process is highly compromised in the presence of TNF alpha. On the other hand, in the presence of ROCK inhibitor the adipogenic differentiation occurs as previously observed (Figure 5). Moreover, the obtained results, both in the presence TNF alpha and ROCK inhibitor in the GG hydrogel particles, compare to those obtained in standard 2D drug screening culture conditions used as control (Figure 6B). 

## 4. Discussion and Conclusions

The development of adipose tissue equivalents has been relying on different strategies that, despite the promising results, also present some limitations, namely in terms of achieved degree of differentiation and stability of the constructs. In this work, we intended to tackle these limitations by exploring the natural features of GG to recreate stable and reliable adipose-like microtissues. Therefore, we tuned GG hydrogels properties by changing the polymer content generating constructs with Young’s moduli between 2.7 and 12.9 kPa. Formulations with 0.5% and 0.75% of GG showed a modulus in the range of the native adipose tissue that varies between 3 and 7 kPa [25,26,27,28]. In opposition, hydrogel formulations with 1.25% of GG have a modulus of approximately 12kPa, in the highest limit reported in the literature for subcutaneous adipose tissue [28]. The amount of polymer also affected the mass loss profile of the GG particles in an inverse proportion as expected by our previous results [21], although the total amount of mass loss occurs within the first day. This is likely to correlate mostly to the non-polymeric content of GG hydrogel, rather than to the hydrolysis of the polymer. In fact, when the hydrogels are placed in the saline solution an exchange of ions/water from the culture medium, in which the hydrogels were prepared, to the saline solution occurs resulting in mass loss within the first day. This explanation is also consistent with the fact that when the polymeric/non-polymeric ratio is lower (0.5% GG hydrogel particles), the percentage of lost mass is higher due to the higher amount of water coming out from the hydrogels to reach an equilibrium state. Moreover, the absence of hydrolysis and consequently the lack of degradation of the polymer can be confirmed by the dried mass of the hydrogel particles at the end time point, in which the particles with successively lower polymeric content seem to gain weight. This is due to the amount of salts, in addition to the polymeric content that keeps constant, that is kept in the dried structure and that is higher for the formulation with lower polymeric content in which a higher water loss was observed. 

It is also been shown that the mechanical properties of 3D microenvironments influence adipogenic differentiation although differently depending on the use of adipose tissue decellularized [29] or biomaterials-based [7] matrices. We confirmed the adipogenic differentiation of hASCs within the GG hydrogels by assessing the expression profile of early (PPARγ) and late (FABP4) adipogenic markers in the different formulations and along the time. Although the action mechanism of PPARγ is not fully understood, PPARγ is a transcriptional factor that is highly expressed in adipocytes and is a major regulator of adipogenesis through the modulation of other adipogenic-related genes expression, such as FABP4 [30]. Having this in consideration, the upregulation of the expression of this gene as the induction period increases confirmed the triggering of the adipogenic differentiation and its maintenance in induction medium. While we could expect a downregulation after some time of culture, the presence of PPARγ agonist troglitazone in the induction medium might in part be responsible for the observed upregulated expression of PPARγ [31] at latter times. This is also supported by the fact that the level of upregulation, in the condition using induction and maintenance (without troglitazone) media, was lower, particularly for the 0.5% GG hydrogel particles, and comparable to the same time period only in induction medium. The gene expression results showed that FABP4 expression is favoured in hydrogels with lower polymeric content and hence in more compliant hydrogels, matching native tissue values [25,26,27,28], which corroborates with the literature [7,29]. Despite the trend for FABP4 mRNA expression results, the semi-quantitative analysis of the amount of FABP4 protein did not reveal significant differences, which might be associated to protein regulation at the posttranscriptional level [32].

Since the success of soft tissue reconstruction approaches is currently limited by the 40–60% volume retention of autologous adipose tissue, depending on its source [33,34], long-term stability of adipose-like tissues is crucial [35]. Proposed biomaterial-based approaches have been exploring several natural polymers such as collagen and alginate, however their degradability might be too fast. In opposition, GG degradation by hydrolysis, which is associated to long-term stability, represents an advantage over these materials for adipose tissue reconstruction. As mentioned before, adipose-like microtissues can be also of great relevance as in vitro models for drug screening or for unravelling the mechanistic of adipose tissue-associated pathologies. Considering this, we were able to demonstrate that the adipose-like microtissues that we generated are responsive to TNFα and Y-27632 (ROCK inhibitor). Y-27632 is a well-known ROCK kinase inhibitors reported as an enhancer of adipogenesis [36], while TNFα is known as a negative regulator of adipogenesis by preventing the early induction of PPARγ and C/EBPα expression [37,38]. Therefore we were able to confirm that our 3D constructs respond as expected, as shown by the absence presence of neutral intracellular lipids accumulation, respectively in the presence of ROCK inhibitor and TNFα. Moreover, the results of our 3D system compare with those obtained in the standard 2D culture conditions confirming the potential of the developed microtissues to be used as in vitro drug screening platforms.

In conclusion, independently of the mechanical properties of the hydrogels, we developed stable and injectable GG-based adipose-like microtissues that can be considered for soft tissue reconstruction, as well as 3D in vitro tissue analogues for drug screening. 

## Figures and Tables

**Table 1:** Primers sequences.

Gene	Primer Forward (5′–3′)	Primer Reverse (5′–3′)	*T*m (°C) ^1^
*PPARγ*	TGGGTGAAACTCTGGGAGAT (20)	TGGCATCTCTGTGTCAACCA (20)	57.3
*FABP4*	AAACTGGTGGTGGAATGCGT (20)	GCGAACTTCAGTCCAGGTCA (20)	58.4
*GAPDH*	AGCCTCAAGATCATCAGCAA (20)	GTCATGAGTCCTTCCACGAT (20)	56

^1^*T*m—Melting temperature.

## Supplementary Materials

The following are available online at https://www.mdpi.com/2306-5354/5/3/52/s1, Figure S1: Capacity of human adipose-derived stem cells (hASCs) to differentiate in different lineages; Table S1: Phenotypic characterization of human adipose-derived stem cells prior differentiation through flow cytometry.
