# Correction: Lago et al. Generation of Gellan Gum-Based Adipose-Like Microtissues. *Bioengineering* 2018, *5*, 52

**DOI:** 10.3390/bioengineering13090972

**Published:** 2026-08-25

**Authors:** Manuela E. L. Lago, Lucília P. da Silva, Catarina Henriques, Andreia F. Carvalho, Rui L. Reis, Alexandra P. Marques

**Affiliations:** 1 3B’s Research Group—Biomaterials, Biodegradables and Biomimetics, Headquarters of the European Institute of Excellence on Tissue Engineering and Regenerative Medicine, University of Minho, Avepark, Barco, 4805-017 Guimarães, Portugal; manuela.lago@i3bs.uminho.pt (M.E.L.L.); lucilia.silva@i3bs.uminho.pt (L.P.d.S.); catarinamhenriques@ua.pt (C.H.); andreiacarvalho@med.uminho.pt (A.F.C.); rgreis@i3bs.uminho.pt (R.L.R.); 2 ICVS/3B’s—PT Government Associate Laboratory, 4806-909 Braga/Guimarães, Portugal; 3 The Discoveries Centre for Regenerative and Precision Medicine, Headquarters at University of Minho, 4704-553 Guimarães, Portugal

In the original publication [1], errors were identified in Figures 4B and 5 involving the duplication of image panels. In Figure 4B, the panels corresponding to the 0.75% polymer condition at day 3 and at day 6 were found to be the same image, despite representing different time points during adipogenic differentiation. Similarly, in Figure 5, two panels representing the 1.25% polymer condition at day 21 and after six days in induction plus nine days in maintenance media were also found to be duplicated.

The corrected Figure 4B and Figure 5 appear below. These errors were limited to figure assembly and do not affect the underlying data, analysis, or interpretation. The corrected images are consistent with the reported results, and therefore, the scientific conclusions of the study remain unchanged. This correction was approved by the Academic Editor. The original publication has also been updated.

## Figures and Tables

**Figure 4:**
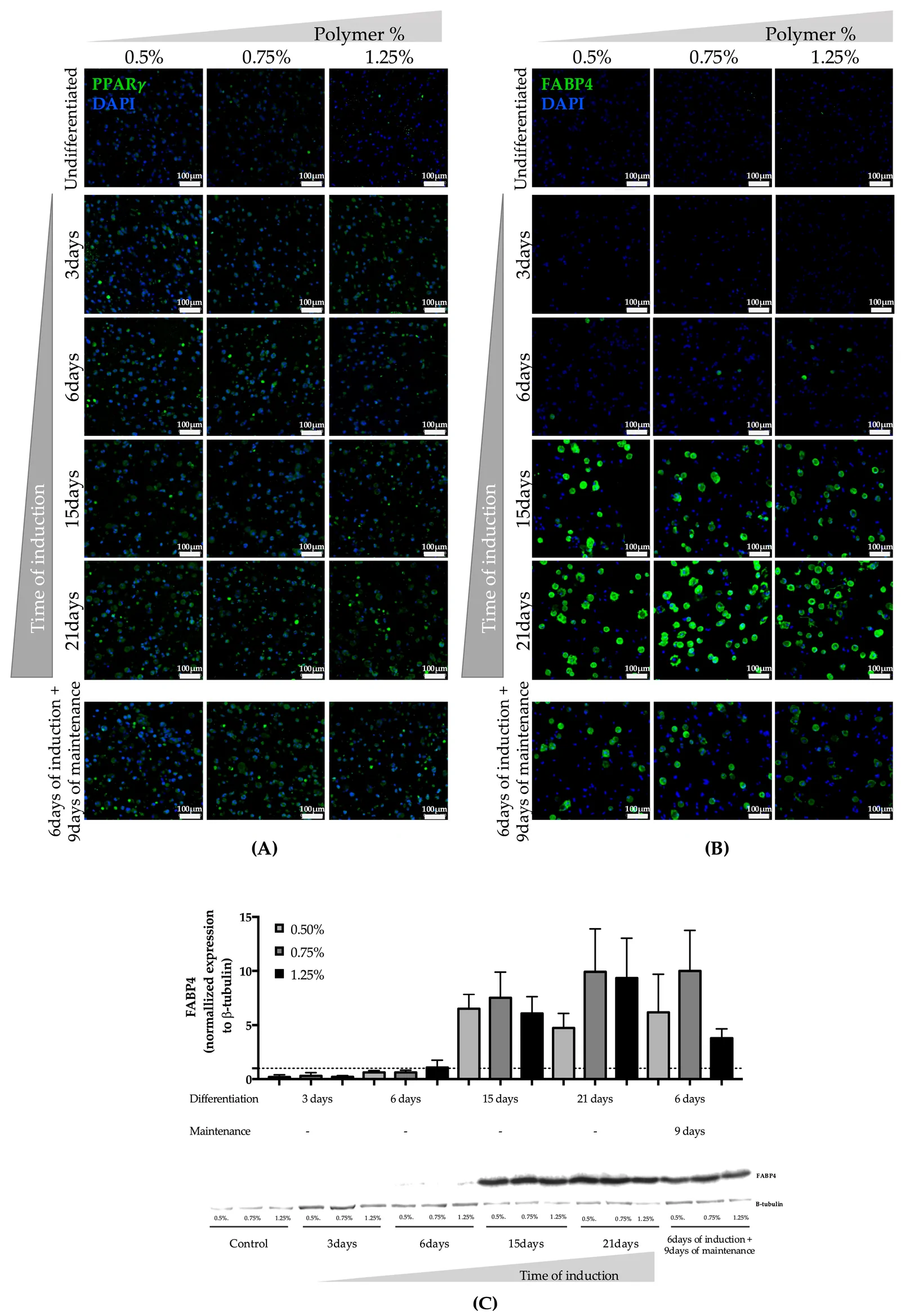
Adipogenic differentiation of human adipose-derived stem cells within gellan gum hydrogel particles with different amounts of polymer along the culture in adipogenic induction medium and after being cultured for six days in induction plus nine days in maintenance media. Representative confocal images of (**A**) PPARγ and (**B**) FABP4 expression (green). Cell nuclei were labelled with DAPI (blue). (**C**) Representative Western blot analysis of the expression of FABP4. Plotted data was normalized against beta-tubulin expression, which was used as a loading control. Scale bar: 100 µm.

**Figure 5:**
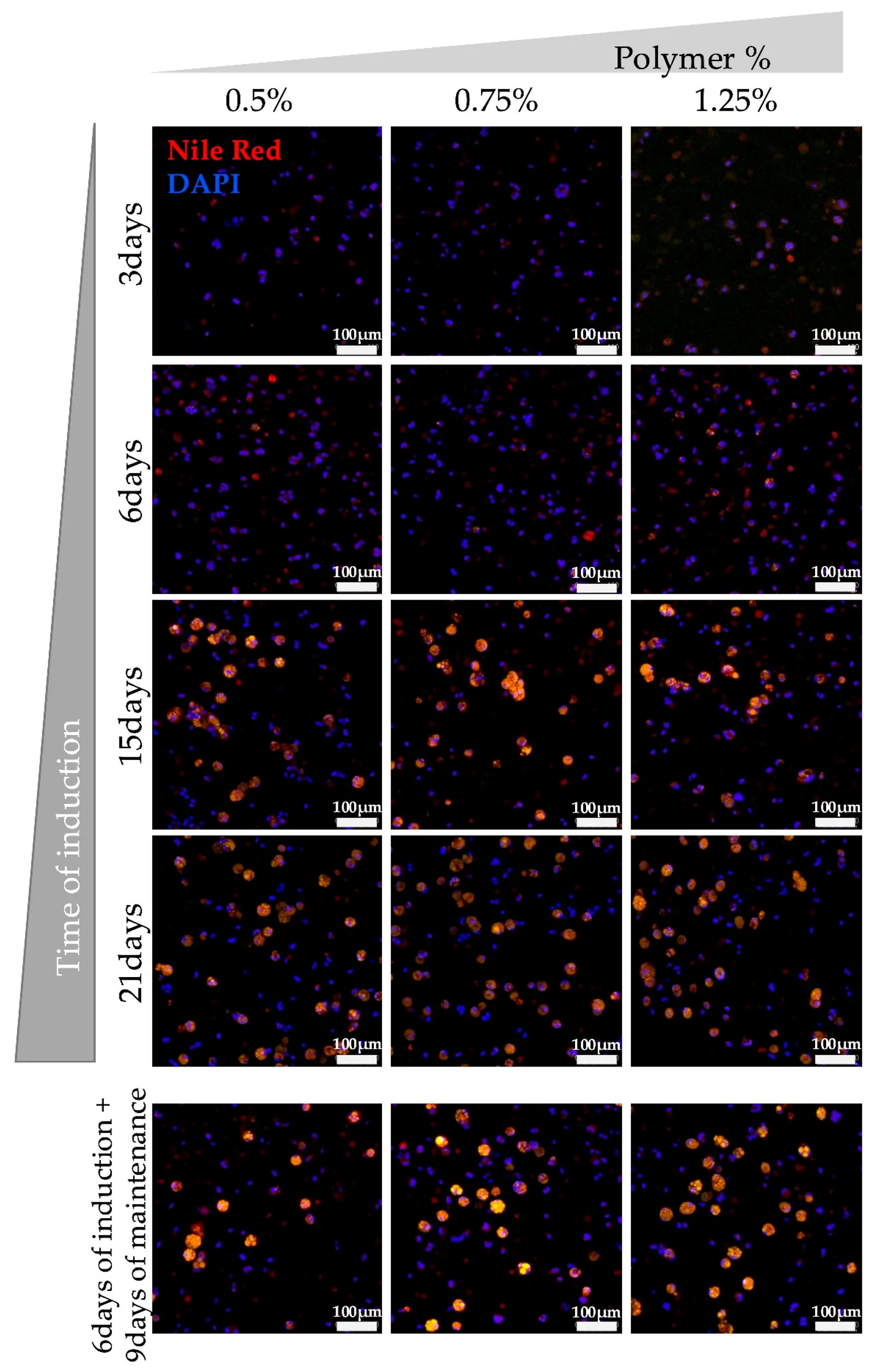
Neutral intracellular lipid accumulation in human adipose-derived stem cell-derived adipocytes within gellan gum hydrogel particles with different amounts of polymer along the culture in adipogenic induction medium and after being cultured for six days in induction plus nine days in maintenance media. Representative confocal images of cells stained with Nile red (yellow). Nuclei were labelled with DAPI (blue). Polar cell membrane lipids are stained in red. Scale bar: 100 µm.
