# Supplementary material for: Generation of Gellan Gum-Based Adipose-Like Microtissues
Source: Bioengineering (Basel). 2018 Jun 27;5(3):52. doi: 10.3390/bioengineering5030052 (PMC6163196; doi:10.3390/bioengineering5030052)
Supplement: Supplementary file 1 [file bioengineering-05-00052-s001.zip › bioengineering-304537-supplementary.pdf]

bioengineering

Supplementary Material

Generation of Gellan Gum-Based Adipose-Like Microtissues

Manuela E. L. Lago [1,2,3], Lucília P. da Silva [1,2], Catarina Henriques [1,2], Andreia F. Carvalho [1,2], Rui L. Reis [1,2,3] and Alexandra P. Marques [1,2,3,*]

[1] 3B's Research Group—Biomaterials, Biodegradables and Biomimetics, Headquarters of the European Institute of Excellence on Tissue Engineering and Regenerative Medicine, University of Minho, Avepark, Barco, 4805-017 Guimarães, Portugal; manuela.lago@i3bs.uminho.pt (M.E.L.L.); lucilia.silva@i3bs.uminho.pt (L.P.d.S.); catarinamhenriques@ua.pt (C.H.); andreiacarvalho@med.uminho.pt (A.F.C.); rgreis@i3bs.uminho.pt (R.L.R.);

[2] ICVS/3B's—PT Government Associate Laboratory, Braga/Guimarães, Portugal

[3] The Discoveries Centre for Regenerative and Precision Medicine, Headquarters at University of Minho, Guimarães, Portugal

* Correspondence: apmarques@i3bs.uminho.pt

Table S1. Phenotypic characterization of human adipose-derived stem cells prior differentiation through flow cytometry.

	Markers (%)		
	CD105	CD90	CD73
Population 1	99,62	99,00	99,82
Population 2	99,91	99,97	99,91
Population 3	99,94	99,98	99,86

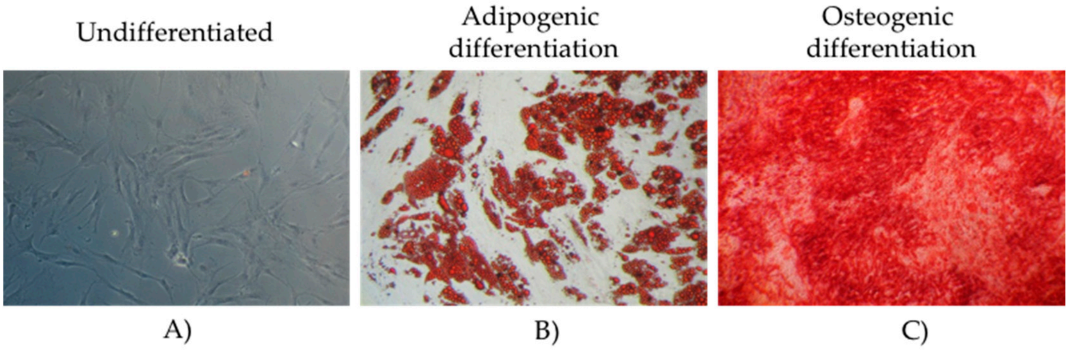

Figure S1. Capacity of human adipose-derived stem cells (hASCs) to differentiate in different lineages. Undifferentiated hASCs **(A)**, and respective differentiation in an adipogenic lineage.**(B)**, as shown by the lipidic staining with Oil Red O, as well differentiation in an osteogenic lineage.**(C)**, as shown by the staining with alizarin red.
